# ﻿A new *Dichocarpum* W.T.Wang & P.K.Hsiao (Ranunculaceae) species from Guizhou, China

**DOI:** 10.3897/phytokeys.262.141266

**Published:** 2025-08-26

**Authors:** Bo Wang, Ze-Huan Wang, Wen-Fen Xu, Yun-Chao Li, Xiang Lu, Qing-Wen Sun, Shun-Zhi He

**Affiliations:** 1 Pharmacognosy Laboratory, College of Pharmacy, Guizhou University of Traditional Chinese Medicine, Guiyang 550025, Guizhou, China Guizhou University of Traditional Chinese Medicine Guiyang China; 2 Department of Traditional Chinese Medicine, Guizhou Institute for food and drug control, 550004, Guiyang, China Guizhou Institute for food and drug control Guiyang China

**Keywords:** China, *
Dichocarpum
*, new species, taxonomy

## Abstract

A new species of *Dichocarpum* from Guizhou, China, is described and illustrated. Morphologically, it resembles *D. auriculatum*, *D. trifoliolatum*, *D. basilare*, *D. franchetii*, *D. arisanense*, *D. adiantifolium*, and *D. uniflorum*. However, it differs from these seven species by its creeping stolons, 3–5-foliolate leaflets with scalloped or suborbicular shape, and an inflorescence consisting of a single flower. The phylogenetic relationship with other species of the genus is examined based on three chloroplast gene segments (*matK, trnL-F, trnH-psbA*) and one nuclear ribosomal DNA region (ITS). In addition, an identification key to all Chinese species of *Dichocarpum* is provided.

## ﻿Introduction

The genus *Dichocarpum* W. T. Wang & P. K. Hsiao (9: 323, 1964) was originally described by W. T. Wang and P. K. Hsiao, encompassing 16 species. These species can be unambiguously distinguished from *Isopyrum* L. (2: 245, 1742), which was initially defined by Linnaeus, based on several key characteristics. These include trifoliate or pedate compound leaves, petals that are non-saccate and possess slender stalks at their base, two carpels, follicles that are Y-shaped and fused at the base, and seeds that are predominantly round and brown.

Following the rigorous taxonomic revisions carried out by Fu (1988) and Tamura (1995), in conjunction with the comprehensive molecular phylogenetic studies conducted by Jiang et al. (2015), Xiang et al. (2017), and Nguyen et al. (2020), and the inclusion of six newly reported species (*D. malipoenense*, *D. lobatipetalum*, *D. wuchuanense*, *D. hagiangense*, *D. uniflorum*, and *D. yongshanense*) as reported by Tao (1989), Wang and Liu (2015), Jiang et al. (2015), Nguyen et al. (2020), Ying (2023), and Li et al. (2024) – with two of them (*D. malipoenense* and *D. lobatipetalum*) being synonyms of *D. hypoglaucum* (Xie et al. 2017) – *Dichocarpum* currently encompasses 22 accepted species.

These species are primarily distributed in subtropical forests of eastern Asia, with a few extending to warm and temperate forests. Specifically, 12 species are found in China, one is endemic to the eastern Himalayas, three are distributed in Vietnam, and eight are present in Japan (Hsiao and Wang 1964; Fu 1988; Tamura 1995; Fu and Robinson 2001; Jiang et al. 2015; Wang and Liu 2015; Xiang et al. 2017; Nguyen et al. 2020; Ying 2023; Li et al. 2024).

In July 2018, during a field expedition to Longli County, Guizhou Province, a unique specimen of *Dichocarpum*, characterized by stolons and fan-shaped leaflets, was collected. Returning to the same locality on 25 March 2022, we observed the previous population in full bloom, displaying a solitary white flower. Through rigorous morphological analysis, an extensive literature review (Hsiao and Wang 1964; Fu 1988; Tao 1989; Tamura 1995; Fu and Robinson 2001; Jiang et al. 2015; Wang and Liu 2015; Xiang et al. 2017; Nguyen et al. 2020; Ying 2023; Li et al. 2024), and thorough verification of specimens (https://www.cvh.ac.cn/), we carefully compared this species with all other known *Dichocarpum* species in China and neighboring countries. As a result, we have confirmed that this specimen represents a suspected new species.

## ﻿Material and methods

### ﻿Morphological study

The morphological data for the putative new species were primarily obtained from direct observations of living plants. Additionally, select living specimens were preserved as herbarium specimens and deposited at the Guizhou Herbarium of Traditional Chinese Medicine (GZTM). For further validation, we meticulously compared our observations with descriptions in the existing literature (Hsiao and Wang 1964; Fu 1988; Tao 1989; Tamura 1995; Fu and Robinson 2001; Jiang et al. 2015; Wang and Liu 2015; Xiang et al. 2017; Nguyen et al. 2020; Ying 2023; Li et al. 2024) and cross-referenced related *Dichocarpum* specimens through the Chinese Virtual Herbarium (https://www.cvh.ac.cn/), JSTOR (https://plants.jstor.org/), and the Muséum national d’Histoire naturelle (https://science.mnhn.fr/institution/mnhn/search).

### ﻿Molecular phylogenetic analysis

#### ﻿Total DNA extraction, PCR amplification, and sequencing

The total DNA was extracted from silica gel–dried tissue utilizing the TSINGKE Plant DNA Extraction Kit (Universal). Four distinct DNA regions were selected for analysis: three chloroplast DNA (cpDNA) regions — *matK*, *trnL-F*, and *trnH-psbA* — as well as one nuclear ribosomal DNA region, the internal transcribed spacer (ITS). Amplification of these four DNA regions was achieved through standard polymerase chain reaction (PCR). Specifically, the *matK*, *trnH-psbA*, *trnL-F*, and ITS regions were amplified and sequenced using the following universal primer pairs: *matK*-AF2 forward (5’-CTT TCA GGA RTA CAT TTA TGC-3’) and *matK*-8R2 reverse (5’-ACG WGC CAA AGT TCT AGC AC-3’) (Wang et al. 2007); *trnH*^GUG^ (5’-CGC GCA TGG TGG ATT CAC AAT CC-3’) (Tate and Simpson 2003) and *psbA* (5’-GTT ATG CAT GAA CGT AAT GCT C-3’) (Sang et al. 1997); *trnL-F* c (5’-CGA AAT CGG GTA GAC GCT ACG-3’) and *trnL-F* f (5’-ATT TGA ACT GGT GAC ACG AG-3’) (Taberlet et al. 1991); and ITS1 (5’-GGA AGT AAA AGT CGT AAC AAG G-3’) and ITS4 (5’-TTA TTG ATA TGC TTA AAC TCA GCG GG-3’) (White et al. 1990).

The PCR products were visualized via agarose gel electrophoresis, stained with bromophenol blue, and observed through a Gel Doc system. The resulting products were then sequenced on an Applied Biosystems 3730XL sequencer. The four sequences were deposited in GenBank (http://www.ncbi.nlm.nih.gov) with the accession numbers PP990698, PQ041823, PQ041855, and PQ041882.

#### ﻿Phylogenetic reconstruction

In this study, a total of 124 DNA sequences were examined, consisting of four newly sequenced DNA samples and 120 sequences obtained from the studies of Xiang et al. (2017) and Nguyen et al. (2020), which collectively represent 35 species. Vouchers and GenBank accession numbers are listed in Table 1. To ensure the integrity and accuracy of the data, the resulting sequences were initially screened against the GenBank database (https://www.ncbi.nlm.nih.gov) using BLAST to assess potential contamination and verify the intended target markers. Alignment of the sequences was performed using MAFFT v7.505 (Katoh and Standley 2013). Next, we used Gblocks 0.91b (Talavera and Castresana 2007) within PhyloSuite v1.2.3 (Zhang et al. 2020) to adjust the alignments and used its Concatenate Sequence function to connect the sequences.

**Table 1.** The taxa employed in this study, voucher details, and GenBank accession numbers. All the information is derived from Jiang et al. (2015), Xiang et al. (2017), and Nguyen et al. (2020).

Taxon	Voucher	Locality	Altitude (m)	matK	trnH-psbA	trnL-F	ITS
*Dichocarpum adiantifolium*	Anonymous 196976 (PE)	Sikkim	2000–2850	–	KY235719	KY235746	KY235684
*Dichocarpum arisanense*	Chen et al. 20110365 (PE)	Taiwan	1200–2600	KY235700	KY235717	KY235744	KY235682
*Dichocarpum arisanense*	Zhong SW 450 (TAIF)	Taiwan	–	KY235701	KY235718	KY235745	KY235683
*Dichocarpum auriculatum*	Jin & Zhou 2009041412 (GZTM)	Sichuan	650–1600	JN605363	HQ844054	–	HQ727692
*Dichocarpum auriculatum*	Zhu et al. 2755 (PE)	Sichuan	–	KY235703	KY235721	KY235748	KY235686
*Dichocarpum basilare*	Jin & Zhou 2009041018a (GZTM)	Guizhou	550	JN605364	–	–	HQ844055
*Dichocarpum carinatum*	Fu DZ 84332 (PE)	Sichuan	500–700	KY235704	KY235723	KY235749	KY235688
*Dichocarpum dalzielii*	Wang W 111 (PE)	Chongqing	750–1600	EF437130	KY235724	EF437098	EF437115
*Dichocarpum dalzielii*	Tan CM 9604084 (TAIF)	Jiangxi	–	KY235705	KY235725	KY235750	–
*Dichocarpum fargesii*	QL-152 (PE)	Shanxi	1300–1600	KY235706	KY235726	KY235751	KY235689
*Dichocarpum fargesii*	Yan & Zhou 1055743 (GZTM)	Guizhou	–	JN605366	HQ844058	–	HQ844049
*Dichocarpum franchetii*	Anonymous 360 (KUN)	Guangxi	900–2000	KY235707	KY235727	KY235752	KY235690
*Dichocarpum franchetii*	Anonymous 732 (PE)	Chongqing	–	KY235708	KY235728	KY235753	KY235691
*Dichocarpum hypoglaucum*	Fu DZ 84329 (PE)	Yunnan	1250	KY235709	KY235729	KY235754	KY235692
*Dichocarpum lobatipetalum*	Shui et al. 20300 (PE)	Yunnan	1700	KY235710	KY235730	KY235755	KY235693
*Dichocarpum nipponicum*	Kosuge 556 (PE)	Japan	200–1900	KY235711	KY235731	KY235756	KY235694
Dichocarpum nipponicum var. sarmentosum	Nagamsn 4507 (HAST)	Japan	350–800	KY235712	KY235732	KY235757	KY235695
*Dichocarpum pterigionocaudatum*	Kosuge 064 (PE)	Japan	400–820	–	KY235733	–	–
*Dichocarpum stoloniferum*	Togashi s.n. (HAST)	Japan	1400–1800	–	KY235734	KY235758	KY235696
*Dichocarpum stoloniferum*	Murata & Chen 9697 (KUN)	Japan	–	KY235713	KY235735	KY235759	KY235697
*Dichocarpum sutchuenense*	Wang W 69 (PE)	Guizhou	1450–2150	KY235714	KY235736	KY235760	EF437116
*Dichocarpum trachyspermum*	Kosuge 963 (PE)	Japan	70–500	KY235715	–	KY235761	KY235698
*Dichocarpum trifoliolatum*	Jin & Zhou 2009040910a (GZTM)	Sichuan	800	JN605369	–	–	HQ844062
*Dichocarpum wuchuanense*	Anonymous s.n. (PE)	Guizhou	650–1000	–	KY235722	–	KY235687
*Dichocarpum hagiangense*	Trinh NB & Pham VT VNM-VNM00023655 (HNU)	Vietnam	1297	–	–	–	MT739412
*Dichocarpum longliense*	Bo Wang wb-202203022 (GZTM)	Guizhou	1139	PQ041823	PQ041855	PQ041882	PP990698
*Aquilegia oxysepala*	Chen et al. s.n. (PE)	Jilin	–	EF437127	KY235737	EF437096	EF437114
*Enemion raddeanum*	Chen & Xu 2090 (PE)	Jilin	–	EF437131	KY235738	EF437100	EF437117
*Isopyrum manshuricum*	Wang W LN004 (PE)	Liaoning	–	EF437133	KY235739	EF437102	EF437119
*Leptopyrum fumarioides*	Guo CC 20080422 (PE)	Jilin	–	KY235716	KY235740	KY235762	KY235699
*Paropyrum anemonoides*	Wundish U. 177 (PE)	Xinjiang	–	EF437132	–	EF437101	EF437118
*Paraquilegia microphylla*	Li CY 001 (PE)	Chongqing	–	EF437136	–	EF437105	EF437122
*Semiaquilegia adoxoides*	Shao Q 02 (PE)	Hunan	–	EF437137	KY235741	EF437106	EF437123
*Thalictrum robustum*	Wang W 38 (PE)	Guizhou	–	EF437138	KY235742	KY235763	EF437125
*Urophysa henryi*	Wang W 96 (PE)	Guizhou	–	EF437139	KY235743	EF437109	EF437126

Subsequently, the index of substitution saturation (Iss) was calculated using DAMBE v5.3.19 (Xia 2013), resulting in Iss values that were significantly less than Iss.c, with a significance level of P = 0.0000. This indicated that the data were suitable for phylogenetic analysis. Phylogenetic analyses involving cpDNA (*matK*, *trnL-F*, and *trnH-psbA*) and ITS datasets were performed using the maximum likelihood (ML) method in IQ-TREE 2.4.0 (Nguyen et al. 2015) and the Bayesian inference (BI) approach in MrBayes v3.2.7a (Ronquist et al. 2012). We constructed an ML phylogenetic tree in IQ-TREE with the following parameter settings: -m GTR+F+I+G4, -alrt 1000, -bb 1000, -bnni. The BI phylogenetic tree was constructed in MrBayes under the GTR+F+G4 nucleotide substitution model selected by MrModeltest v2.2.0 (Kalyaanamoorthy et al. 2017). Four independent Markov chains were run for a total of 5 million generations, with trees sampled every 1,000 generations. To ensure convergence of the Markov chains, diagnostics were checked using Tracer v1.7.2 (Rambaut et al. 2018), which confirmed that the effective sample sizes (ESS = 4722–8752) for all parameters exceeded 200, indicating adequate convergence. After discarding 25% of the initial samples as burn-in, a consensus tree with posterior probabilities was constructed using the remaining samples. The outgroup selection was based on the reference study by Xiang et al. (2017). Finally, the consensus tree was visualized using the Interactive Tree of Life (iTOL) online platform (https://itol.embl.de/) (Letunic and Bork 2021).

## ﻿Results

Morphological studies have revealed that the suspected new species displays notable differences from existing *Dichocarpum* species in several key characteristics. The leaves of this potential new species are scalloped or suborbicular, which contrasts with the rhombic to broadly rhombic-ovate leaves found in other species. Its inflorescence bears a solitary flower, distinguishing it from other species that typically have inflorescences with three to six flowers. Although *D. uniflorum* also features a single flower, it differs from the suspected new species in terms of leaflet quantity and flower size. Furthermore, the suspected new species possesses stolons, a trait relatively rare within the *Dichocarpum* genus. To date, only *D. stoloniferum*, native to Japan, is known to exhibit stolons. Although both *D. stoloniferum* and the putative new species possess stolons, they exhibit significant differences in petal morphology. Specifically, the petals of *D. stoloniferum* are bilabiate (two-lipped), whereas those of the putative new species are flat and fan-shaped. Table 2 provides a detailed comparative analysis of this species with its close relatives. These thorough morphological comparisons offer a scientific foundation for the identification and classification of the suspected new species.

**Table 2.** Morphological comparison among *Dichocarpum longliense*, *D. trifoliolatum*, *D. basilare*, *D. carinatum*, *D. franchetii*, *D. arisanense*, *D. adiantifolium*, and *D. uniflorum*.

Characters	D. longliense	D. trifoliolatum	D. basilare	D. carinatum	D. franchetii	D. arisanense	D. adiantifolium	D. uniflorum
stolons	creeping stolons	without stolons	without stolons	without stolons	without stolons	without stolons	creeping stolons	creeping stolons
rhizome	1–1.5 cm long, 0.5–0.8 cm in diameter	ca. 16 cm long, ca. 0.4 cm in diameter	ca. 1 cm long, ca. 0.6 cm in diameter	8–10 cm long, 0.5–0.8 cm in diameter	inconspicuous	unknown	unknown	8–9 mm long, 9–10 mm diameter
cauline leaves present	no	no	no	no	no	no	1 or no	no
leaves	petiole 5–11 cm; 3–5-foliolate; lateral leaflets and central leaflet scalloped or suborbicular	petiole 6.2–8.3 cm; simple or-foliolate; lateral leaflets obliquely obovate, central leaflet rhombic-ovate	petiole 2–4.7 cm; (3–)5-foliolate; lateral leaflets obliquely rhombic, central leaflet rhombic to rhombic-obovate	petiole to 12 cm; 12–15-foliolate; lateral leaflets smaller, unequal in size, central leaflet subrhombic to rhombic-ovate	petiole 2.5–7 cm; lateral leaflets subflabellate, obliquely ovate, or subround, central leaflet suborbicular to subflabellate	petiole 3–4 cm; lateral leaflets broadly rhombic to flabellate-obovate, central leaflet flabellate-obovate	petiole 3–4 cm; lateral leaflets obliquely ovate or subcircular, central leaflet flabellate to suborbicular	petiole 3–4.5 cm; leaves 7–9; lateral leaflets suborbicular,central leaflets fan-like
inflorescences	only one flower	3-flowered	3–5-flowered	3–5-flowered	3–7-flowered	few flowered	3–7-flowered	only one flower
sepals	white, ovate-oblong, ca. 15 mm long	pinkish, obovate, ca. 3.5 mm long	unknown	unknown	white, obovate, 3.5–4.5 long,	narrowly ovate, ca. 4 mm long	obovate, ca. 3.5–4.54 mm long	white, oblong to ovate-oblong, 6–7 mm long
petal limbs	fan-shaped, ca. 6 mm, apex concave deficiency	flabellate, ca. 2.5 mm, apex retuse	unknown	unknown	suborbicular, 1–1.2 mm, apex retuse or entire	orbicular, ca. 1 mm	subcircular, 1–1.2 mm, slightly concave or entire	clavate, 1mm long
stamens	25–40	unknown	unknown	unknown	unknown	5 or 10	unknown	12–15
follicle	linear, ca. 10 mm	linear, 8–10mm	linear, 7.5–10 mm	linear, ca. 10 mm	linear, 7–10 mm	linear, ca. 9 mm	linear, 7–10 mm	slender, 4–5 mm
seed	unknow	ellipsoid, ca. 2.5 mm in diameter	subglobose, ca. 1.5 mm in diameter	subglobose, ca. 1 mm in diameter	globose, ca. 1 mm in diameter	globose, ca. 0.75 mm in diameter	globose, yellowish-brown, ca. 1 mm in diameter	ellipsoid to ovoid, 1.5–1.6 mm in diameter

**Figure 1.**
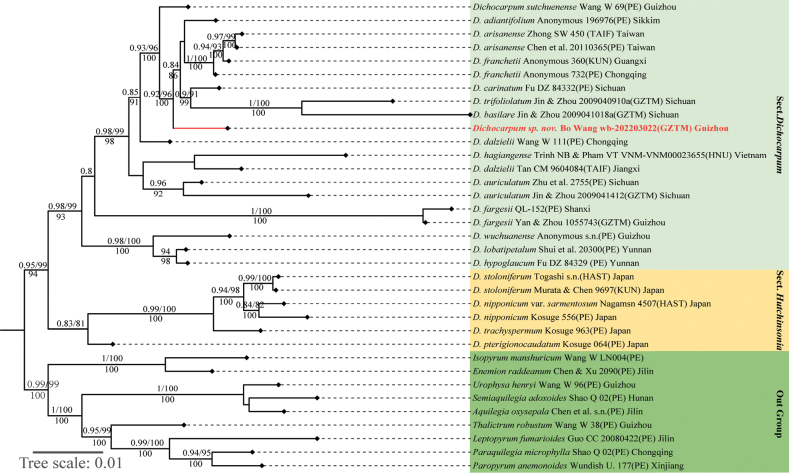
ML tree inferred from the combined cpDNA and ITS data. Numbers above and below branches are bootstrap values (SH-aLRT ≥ 0.8, UFBoot ≥ 80) and Bayesian posterior probabilities (PP ≥ 80), respectively.

In our comprehensive phylogenetic analysis, we constructed a robust phylogenetic tree using both maximum likelihood (ML) and Bayesian inference (BI) methods, based on combined cpDNA and nrITS datasets (Fig. 1). The ML and BI analyses produced congruent results, with identical tree topologies. Our findings reveal that *Dichocarpum* is monophyletic, supported by strong statistical evidence, and we identified two major clades represented by sect. Hutchinsonia and sect. Dichocarpum, with high bootstrap support (SH-aLRT = 0.95, UFBoot = 99) and Bayesian posterior probability (PP = 94). This phylogenetic framework provides critical insights into the complex evolutionary relationships among the various taxonomic groups within *Dichocarpum*. A particularly noteworthy outcome of our study is the robust placement of the potential new species (*Dichocarpum* sp.) within sect. Dichocarpum. Specifically, this species is firmly nested within this section and forms a sister-group relationship with a clade that includes *D. basilare*, *D. trifoliolatum*, *D. carinatum*, *D. franchetii*, *D. adiantifolium*, and *D. arisanense*. This placement is supported by a moderate bootstrap value (SH-aLRT = 0.92, UFBoot = 96) and a high Bayesian posterior probability (PP = 100), indicating a high level of confidence in the phylogenetic positioning of this potential new species.

### ﻿Conclusion

Based on the morphological and molecular differences discussed above, we conclude that the specimen from Longli County represents a new species of *Dichocarpum*. Below is the formal description of the new species.

### ﻿Taxonomic treatment

#### ﻿ Dichocarpum longliense

Taxon classificationPlantaeRanunculalesRanunculaceae

Bo Wang, Q.W.Sun & S.Z.He sp. nov.

F27611A2-C2F5-5DBC-A467-C6E8981E10AD

urn:lsid:ipni.org:names:77368250-1

Figs 2 , 3 , 4

##### Type.

China • Guizhou Province, Longli County, Wantanhe Township, Kaika Village, Kaoru Mountain, 26°19′31.18″N, 106°59′51.60″E, elev. ca. 1139 m, 25 March 2022, *Bo Wang, wb-202203022* (holotype: GZTM!; isotypes: GZTM!).

##### Diagnosis.

This species is similar to *D. basilare*, *D. trifoliolatum*, *D. carinatum*, *D. franchetii*, *D. adiantifolium*, and *D. arisanense*. However, it differs from these species in several key morphological traits. Specifically, its leaflets are scalloped or suborbicular (vs. rhombic to broadly rhombic-ovate in the other six species), and its inflorescence bears only a single flower (vs. 3–7 flowers in the inflorescences of the other species). Additionally, it differs from *D. uniflorum*, which also features a single flower, in terms of leaflet quantity and flower size.

**Figure 2.**
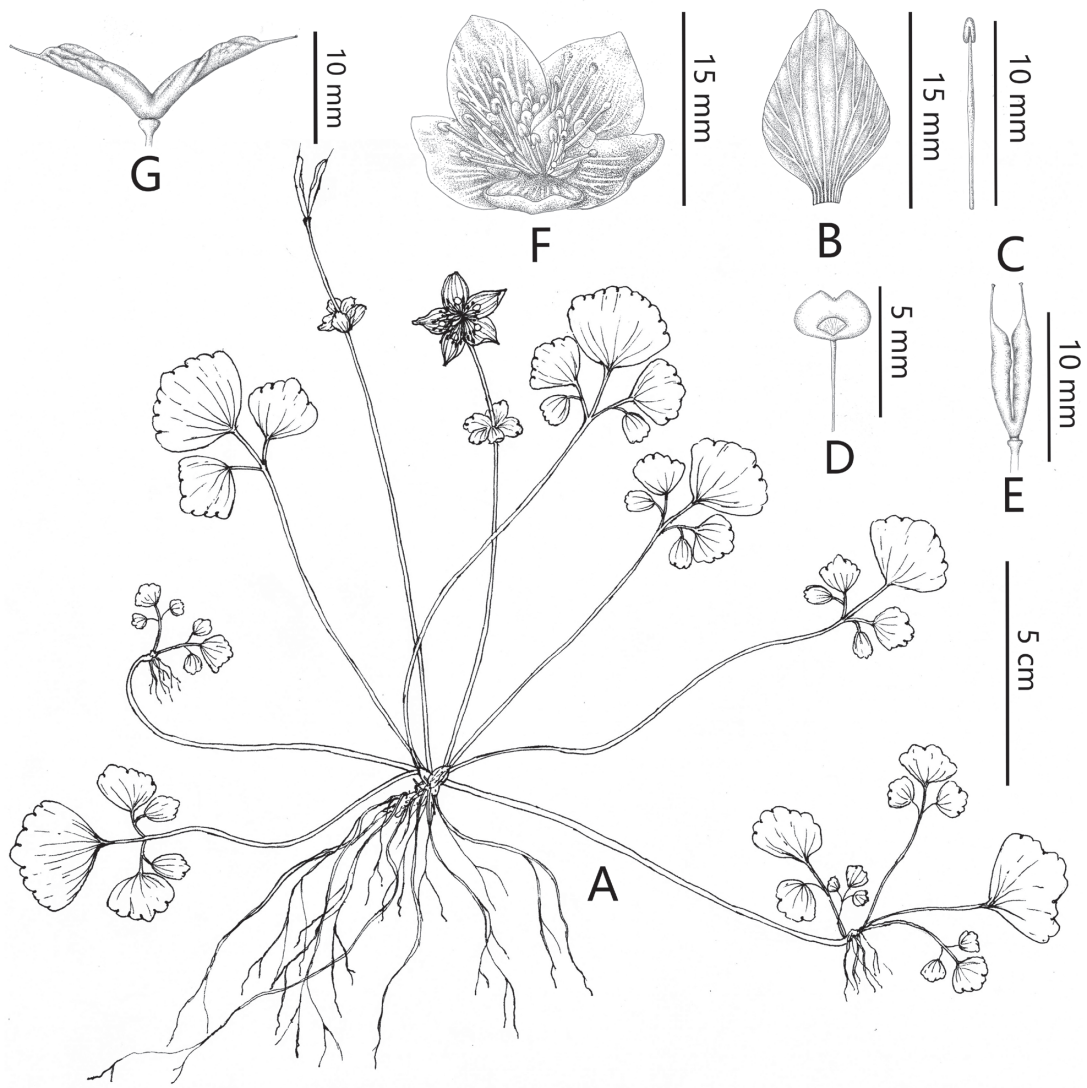
*Dichocarpum longliense*. A. Habitat; B. Sepals; C. Stamens; D. Petal; E. Ovary; F. Flower; G. Fruits. Drawing by Xiang Lu and Na-na Xu from the holotype.

##### Description.

Perennial herb, glabrous. Creeping growth, stolons 2–13 cm long. Rhizome slender, 1–1.5 cm long, ca. 0.5–0.8 cm in diameter. Unbranched, with numerous fibrous roots, 8–14 cm long. Basal leaves 3–8, pedately compound, 3–5-foliolate, thin papery, petiole 5–11 cm, leaf blade 1.5–2.6 × 1–2.4 cm, central leaflet scalloped to nearly rounded, base broadly cuneate or rounded, 2–2.6 × 1.8–2.4 cm, margin distally crenate, apex obtuse, leaflet petiole 0.3–2 cm; lateral leaflets 1–2, scalloped to nearly rounded, 1.5–2 × 1–1.8 cm, margin distally crenate, apex obtuse; without cauline leaves. Bracts 2, opposite, sessile or shortly petiolate, 3-foliolate. Flowering stem ca. 10–15 cm tall. Inflorescence only 1 flower. Sepals 5, ovate-oblong, white, 1.2–1.4 × 0.6–0.8 cm, apex acute. Petal fan-shaped, golden yellow, apex concave deficiency, 6 × 2 mm, claw ca. 2 mm. Stamens 25–40, anther narrowly oblong, ca. 0.8 mm. Filaments ca. 0.9 cm; ovary narrowly obovate, 0.8–1 cm, persistent styles ca. 2 mm. Follicles linear, ca.10 mm, beaks ca. 2–2.5 mm.

**Figure 3.**
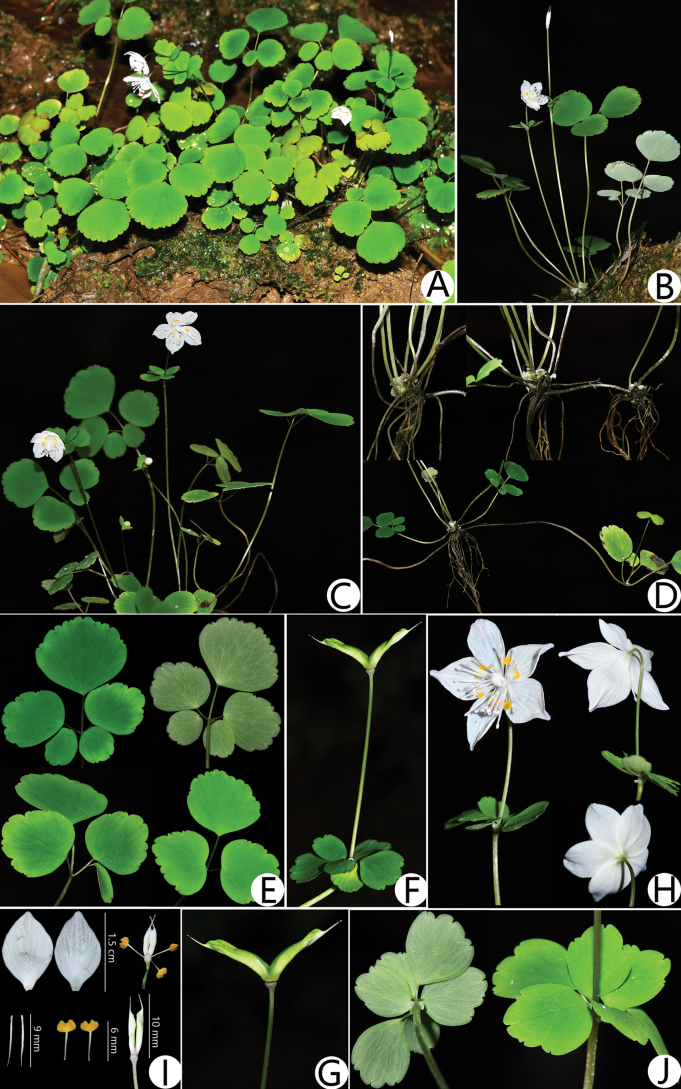
*Dichocarpum longliense*. A–C. Habitat; D. Stolons; E. Leaves; F, G. Fruits; H. Flowers; I. Flower anatomy; J. Bracts. Photographs by Bo Wang.

##### Distribution and ecology.

The new species is currently restricted to the type locality, specifically Kaoru Mountain, located in Kaika Village, Wantanhe Township, Longli County, Guizhou, China, at an approximate elevation of 1139 meters. Here, only three populations comprising approximately 120 individuals were discovered growing in a moist gully and the entrance of a cave. These plants were accompanied by *Adiantum capillus-veneris* Linnaeus (Pteridaceae), *Hymenasplenium* sp. (Aspleniaceae), *Elatostema* sp. (Urticaceae), and other species.

**Figure 4.**
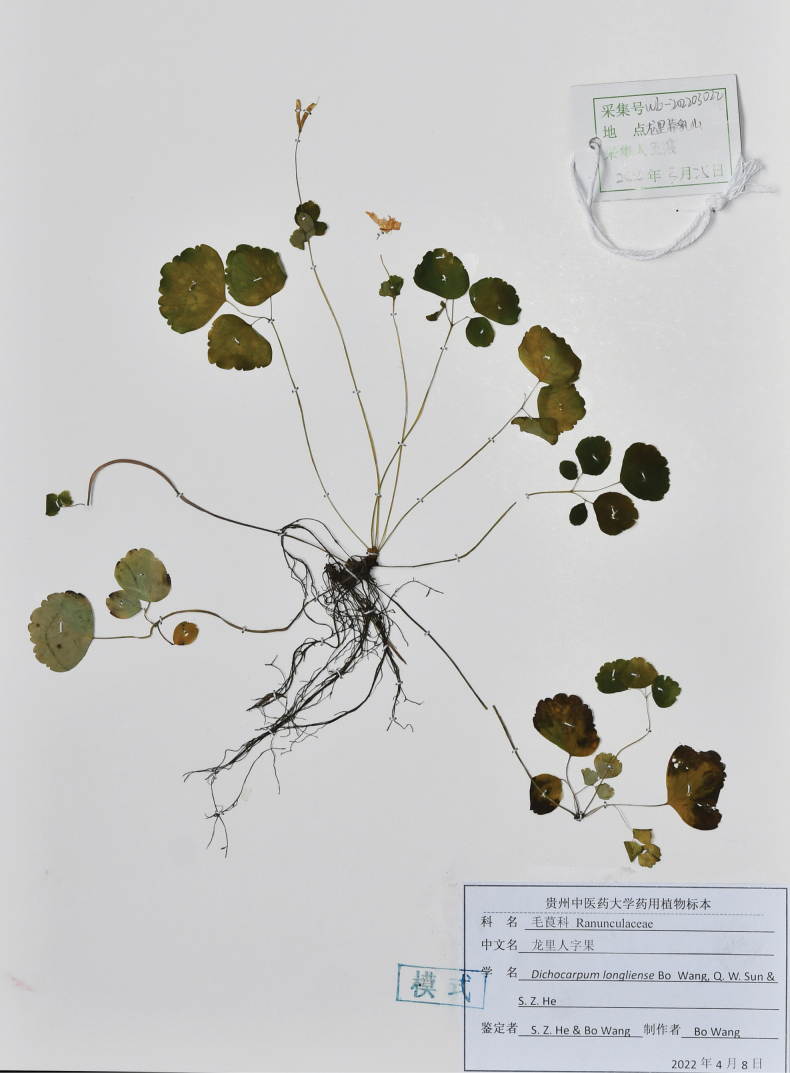
Holotype of *Dichocarpum longliense* (GZTM!).

##### Phenology.

Flowering from March to April; fruiting from April to June.

##### Etymology.

The specific epithet ‘longliense’ is derived from the type locality, Longli (Guizhou, China).

##### Vernacular name.

Simplified Chinese: 龙里人字果; Chinese Pinyin: LóngLǐ RénZìGuǒ.

##### Additional specimen studied (paratype).

China, Guizhou, Guiyang city, Guizhou University of Traditional Chinese Medicine Germplasm Garden, cultivated, 25 March 2022, wb-202203024, introduced by Bo Wang from the type locality.

### ﻿Key to the 15 species and one variety of *Dichocarpum* in China

**Table:** 

1a	Petals funnel-shaped; seeds ovoid-fusiform	***D. fargesii***
1b	Petals flat; seeds globose to subglobose, rarely ellipsoid	2
2a	Stem leaves present	3
3a	Central leaflet rhombic to broadly rhombic-ovate, 1.8–6 × 1.5–5 cm	4
4a	Plant glabrous (without hairs)	***D. auriculatum***
4b	Abaxial surface of leaves covered with short soft hairs	**D. auriculatum var. puberulum**
3b	Central leaflet suborbicular, suborbicular-obovate, flabellate, or flabellate-obovate, 0.5–2.3 × 0.6–2.5 cm	5
5a	Stamens 5 or 10	***D. arisanense***
5b	Stamens 20–45	6
6a	Central leaflet suborbicular-obovate to flabellate-obovate; flower diameter 1.1–2.3 cm	***D. stutchuense***
6b	Central leaflet suborbicular to subflabellate; flower diameter 0.4–0.6 cm	***D. franchetii***
2b	Stem leaves absent	**7**
7a	One flower per plant	**8**
8a	With stolons; 3–5 leaflets	***D. longliense***
8b	Without stolons; 7–11 leaflets	***D. uniflora***
7b	Plants with 2 or more flowers	**9**
9a	3–5 leaflets	**10**
10a	Flower white	***D. wuchuanense***
10b	Flower pinkish-red	**11**
11a	Leaves 3 leaflets or sometimes simple	***D. trifloratum***
11b	Leaves 5 leaflets, rarely 3 leaflets	***D. basilare***
9b	5–15 leaflets	**12**
12a	Rhizome 5–10 cm	**13**
13a	Leaflets with axillary powder; seeds not prominently ridged, smooth	***D. hypoglaucum***
13b	Leaflets without axillary powder; seeds prominently ridged, rough	***D. carinatum***
12b	Rhizome 2–3(–5) cm	**14**
14a	Central leaflet rhombic	***D. dalzielii***
14b	Central leaflet suborbicular, suborbicular-obovate, flabellate-obovate, or subflabellate	**15**
15a	Central leaflet suborbicular-obovate to flabellate-obovate; flower diameter 1.1–2.3 cm	***D. stutchuense***
15b	Central leaflet suborbicular to subflabellate; flower diameter 0.4–0.6 cm	***D. franchetii***

## ﻿Discussion

The discovery of *D. longliense* highlights the rich biodiversity within the genus and underscores the importance of continued exploration. Morphologically, *D. longliense* is distinguished by its scalloped or suborbicular leaflets and solitary flower, features that set it apart from other species with rhombic to broadly rhombic-ovate leaflets and 3–7-flowered inflorescences. These unique traits, along with the presence of stolons, support its classification as a distinct species. Phylogenetically, *D. longliense* is placed within sect. Dichocarpum, forming a sister-group relationship with other well-known species, a positioning supported by both maximum likelihood and Bayesian inference analyses. Given its limited distribution and small population size, *D. longliense* may be vulnerable to environmental changes, emphasizing the need for conservation efforts focused on its unique habitat. Future research should aim to expand the sampling range, assess genetic diversity, and compare this species with its close relatives to further understand its evolutionary significance. Overall, the identification of *D. longliense* not only enriches our understanding of the genus but also highlights the importance of continued exploration and conservation efforts.

## Supplementary Material

XML Treatment for Dichocarpum longliense

## ﻿Additional information

### Use of AI

No use of AI was reported.

### Author ORCIDs

Bo Wang https://orcid.org/0000-0002-1814-3865

Ze-Huan Wang https://orcid.org/0000-0002-7369-5005

Wen-Fen Xu https://orcid.org/0000-0003-1335-2470

Xiang Lu https://orcid.org/0000-0001-9994-0489

Qing-Wen Sun https://orcid.org/0000-0003-0848-5017

Shun-Zhi He https://orcid.org/0000-0002-2082-3181

### Data availability

All of the data that support the findings of this study are available in the main text.
